# Cell-Based NIPT Detects 47,XXY Genotype in a Twin Pregnancy

**DOI:** 10.3389/fgene.2022.842092

**Published:** 2022-03-11

**Authors:** Line Dahl Jeppesen, Tina Duelund Hjortshøj, Johnny Hindkjær, Lotte Hatt, Olav Bjørn Petersen, Ripudaman Singh, Palle Schelde, Lotte Andreasen, Rikke Christensen, Dorte L. Lildballe, Ida Vogel

**Affiliations:** ^1^ ARCEDI, Vejle, Denmark; ^2^ Center for Fetal Diagnostics, Department of Clinical Medicine, Aarhus University, Aarhus, Denmark; ^3^ Department of Clinical Genetics, Copenhagen University Hospital, Rigshospitalet, Copenhagen, Denmark; ^4^ Aagaard Fertility Clinic, Aarhus, Denmark; ^5^ Center for Fetal Medicine, Department of Obstetrics, Copenhagen University Hospital, Rigshospitalet, Copenhagen, Denmark; ^6^ Department of Clinical Medicine, University of Copenhagen, Copenhagen, Denmark; ^7^ Department of Clinical Genetics, Aarhus University Hospital, Aarhus, Denmark; ^8^ Department of Molecular Medicine (MOMA), Aarhus University Hospital, Aarhus, Denmark

**Keywords:** cell-based NIPT, circulating fetal cells, extravillous trophoblasts, cell-free NIPT, sex chromosome anomaly, twin pregnancy, klinefelter syndrome

## Abstract

**Background:** The existing risk of procedure-related miscarriage following invasive sampling for prenatal diagnosis is higher for twin pregnancies and some women are reluctant to test these typically difficultly obtained pregnancies invasively. Therefore, there is a need for noninvasive testing options that can test twin pregnancies at an early gestational age and ideally test the twins individually.

**Case presentation:** A pregnant woman opted for cell-based NIPT at GA 10 + 5. As cell-based NIPT is not established for use in twins, the test was provided in a research setting only, when an ultrasound scan showed that she carried dichorionic twins.

**Materials and Methods:** Fifty mL of peripheral blood was sampled, and circulating fetal cells were enriched and isolated. Individual cells were subject to whole-genome amplification and STR analysis. Three fetal cells were analyzed by chromosomal microarray (aCGH).

**Results:** We identified 20 fetal cells all sharing the same genetic profile, which increased the likelihood of monozygotic twins. aCGH of three fetal cells showed the presence of two X chromosomes and a gain of chromosome Y. CVS from both placentae confirmed the sex chromosomal anomaly, 47,XXY and that both fetuses were affected.

**Conclusion:** NIPT options can provide valuable genetic information to twin pregnancies that help the couples in their decision-making on prenatal testing. Little has been published about the use of cell-based NIPT in twin pregnancies, but the method may offer the possibility to obtain individual cell-based NIPT results in dizygotic twins.

## Introduction

The risk of miscarriage following invasive testing is higher in twin pregnancies compared to singletons (estimated to be 1–2% and <0.5%, respectively) (Sundhedsstyrelsen, 2017; Rose et al., 2020). Increased prevalence of twin pregnancies due to advanced maternal- and paternal age as well as an increased use of assisted reproduction technology (Imaizumi, 1997; Zegers-Hochschild et al., 2009; Chauhan et al., 2010), has therefore made reliable NIPT strategies for twin pregnancies an urgent need in prenatal care.

The vast majority of NIPTs used in both public healthcare systems and by private providers are based on analysis of circulating cell-free fetal DNA (cffDNA) that sheds from the placenta to the maternal blood. We refer to these analyses as cell-free NIPT. Cell-free NIPT covers the common trisomies, sex chromosomal anomalies, and, for some types, specific targets associated with common microdeletions- and microduplications (Dondorp et al., 2015). While cell-free NIPT detects the common trisomies with high accuracy, the test performance is significantly lower for the other chromosomal alterations. In general, cell-free NIPT results must be interpreted with caution because maternal factors such as copy number variants (CNVs), sex chromosomal anomalies (SCAs) and benign- or malignant tumors may interfere with the data interpretation (Lenaerts et al., 2019; Lüthgens et al., 2021).

In contrast to cffDNA or cell-free NIPT, a newly launched test, Evita Test Complete, uses intact circulating fetal cells (extravillous trophoblasts) for detection of aneuploidies and genome-wide copy number variants down to 5 Mb (Kølvraa et al., 2016; Petersen et al., 2017; Vestergaard et al., 2017; Hatt et al., 2020). Extravillous trophoblasts derive from the placental chorionic villi and can only be reliably isolated from maternal blood in the end of first trimester (Hatt et al., 2014; Ravn et al., 2020; E. Davies et al., 2016). We refer to the analysis of circulating fetal cells as cell-based NIPT. Importantly, cell-based NIPT is not affected by maternal factors known to influence the analysis of cffDNA. This was recently demonstrated in a case with a pregnant woman who was mosaic for monosomy X where cell-based NIPT showed an euploid 46,XX female fetus, while cell-free NIPT showed increased risk of 46,X associated with Turner syndrome (Jeppesen et al., 2021). However, more data is needed to determine the sensitivity and specificity before the test is ready for routine clinical use (Vossaert et al., 2019).

All current NIPTs (based on cffDNA or circulating fetal cells) are screening tools and therefore, women who receive a positive NIPT result should be offered follow-up by invasive testing (CVS or AC).

In the present case, we describe how cell-based NIPT (as wells as cell-free NIPT) detected Klinefelter syndrome in a twin pregnancy. Cell-based NIPT gave additional information indicating that this was a monozygotic twin pregnancy, as the genetic profiles of all the isolated cells were identical. This is strongly indicative of both fetuses being affected—a distinction not possible by the cell-free NIPT. This case report demonstrates how noninvasive testing options can provide valuable information to the pregnant couple using the traditional cell-free NIPT and cell-based NIPT, but also how cell-based NIPT may turn out to be particularly useful in twin pregnancies.

## Case Presentation

A 37-year-old pregnant woman (GA 10 + 5) and her partner wanted genetic information about the pregnancy at an early gestational age. They contacted Aagaard Fertility Clinic Denmark, as they offer a commercial cell-based NIPT, EVITA TEST COMPLETE. The pregnancy was naturally conceived. At the clinic, ultrasound examination showed that the pregnancy was a dichorionic twin pregnancy by identification of a “lambda-sign” at the intertwin membrane. The couple was counselled about the limitation of the test, as it was not validated for multiple pregnancies. The couple still opted for cell-based NIPT. Therefore, the test was offered in a research setting.

## Methods

### Cell-Based NIPT

At gestational age 10 + 5, a 50 ml blood sample was collected in five Cell-Free DNA BCT tubes (Streck laboratories, United States). The blood was processed as previously described with slight modifications (Rose et al., 2020). In brief, fetal extravillous trophoblasts were enriched by Magnetic Activated Cell Sorting (Miltenyi Biotech, Germany) using markers and method previously described, and individual cells were isolated by Fluorescence Activated Cell Sorting (FACS) using a BD FACSMelody™ Cell Sorter (BD Biosciences, US). Whole-genome amplification (WGA), identification of fetal cells by short tandem repeat (STR) analysis, CNV analysis by aCGH and data analysis were performed as described before (Sundhedsstyrelsen, 2017). The reference DNA for aCGH was a pool of WGA products of 10 lymphoblastic genomic DNA (Promega, United States), representing an euploid female karyotype (46,XX). The cell-based NIPT aCGH result was analyzed at the Department of Clinical Genetics, Aarhus University Hospital, using the adm-2 algorithm with a 5 Mb threshold for CNV detection as described (Hatt et al., 2020).

### Cell-free NIPT

Twenty mL of blood was sampled in Cell-Free DNA BCT tubes at GA 10 + 5 and the sample was analyzed retrospectively for research purposes. The cell-free NIPT analysis was conducted at the Department of Clinical Genetics, Aarhus University Hospital, as previously described (Sundhedsstyrelsen, 2017).

### Prenatal Diagnosis Using Invasive Sampling

Chorionic villous sampling (CVS) and aCGH were carried out at GA 13 + 1 at the Department of Fetal Medicine and the Department of Clinical Genetics, Rigshospitalet, Copenhagen, Denmark, where the pregnant woman also had her first-trimester screening. Transabdominal approach using a needle guide with separate sampling from each placenta with a 18–20 G double-needle was performed. aCGH was performed using the Agilent SurePrint G3 180K ISCA oligo + SNP array (Technologies, Santa Clara, CA). Labeling and hybridization were performed according to the protocol provided by Agilent.

## Results

**FIGURE 1:**
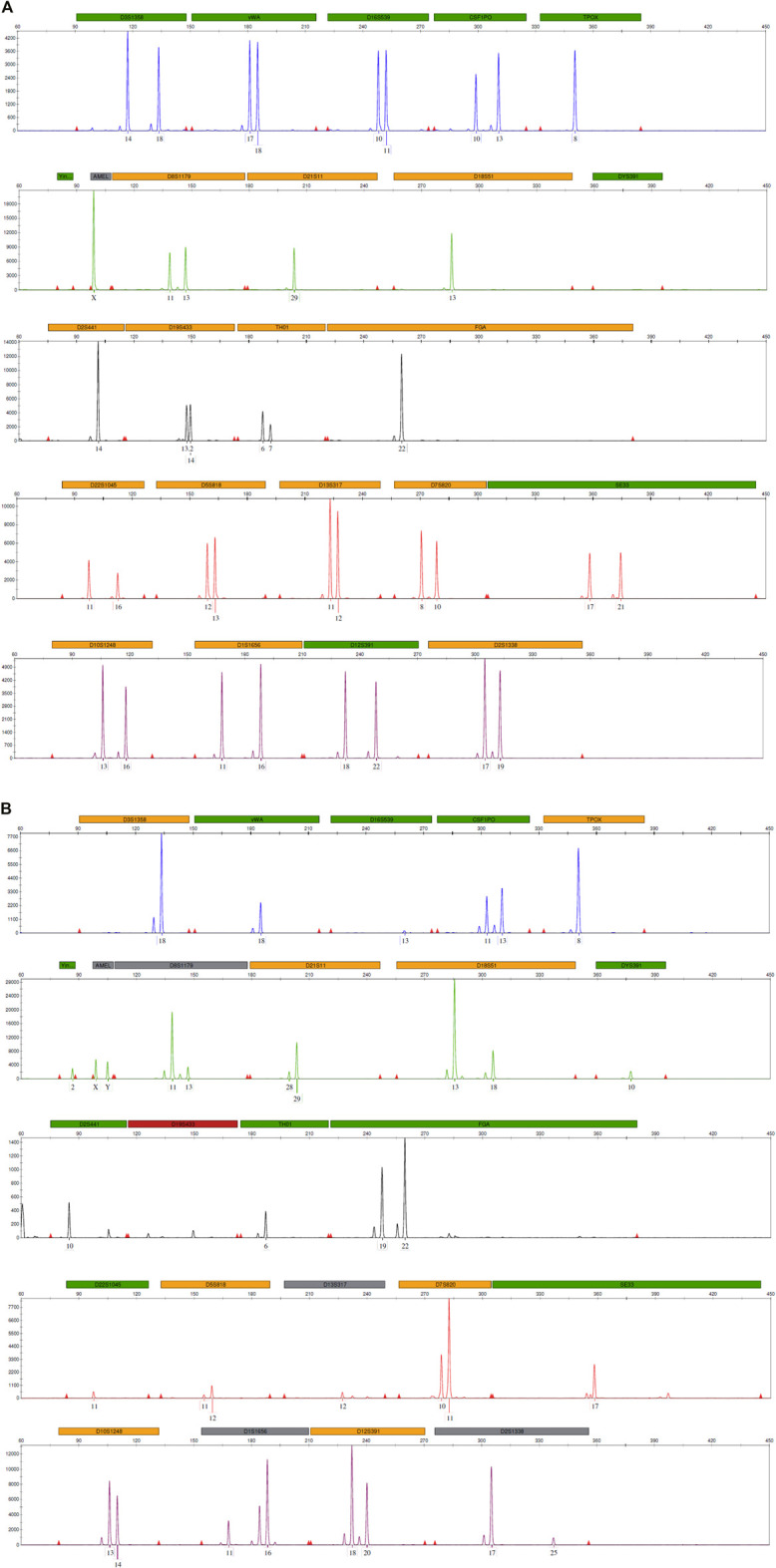
STR profiles from **(A)** maternal DNA and **(B)** a single fetal cell obtained using the GlobalFiler™ PCR amplification kit targeting 24 loci across the human genome, including sex-determining markers, Y indel and Amelogenin. The horizontal panels label the 6-dye, 24-locus STR multiplex after fragment analysis using an ABI3500 genetic analyzer and data analysis using GeneMapper ID-X software (Thermo Fisher Scientific). The marker under each allele peak represents the number of repeats.

For cell-based NIPT, FACS single-cell sorting isolated 32 individual cells, which were subject to WGA and STR analysis. Nine tubes were, however, empty based on DNA concentration measurements following WGA. The STR analysis identified 20 fetal cells, two cells with inconclusive origin due to allele drop-out in the STR analysis and one maternal cell. All 20 fetal cells shared the same STR profile (Figure 1).

**FIGURE 2:**
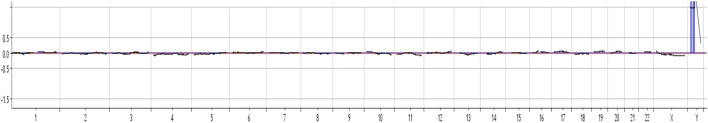
Cell-based NIPT chromosomal microarray of a WGA pool from three fetal cells (GA 10 + 5). The chromosomes (1–22,X,Y) are represented on the horizontal axis and the thresholds for gains and losses are indicated by the vertical axis. The sample is matched against reference DNA representing a female karyotype 46,XX. The result shows presence of two X chromosomes and gain of chromosome Y. Hence, the cell-based NIPT result suggests a 47,XXY karyotype.

A pool of single-cell WGA DNA from three individual fetal cells was analyzed by aCGH. The cell-based NIPT result showed presence of Y-chromosome and two X-chromosomes when compared to female control DNA, Figure 2. This result indicates a fetal 47,XXY karyotype in at least one of the twins, which results in development of Klinefelter Syndrome. No other chromosomal aberrations were detected using a 5 Mb threshold. For research purposes, the three fetal cells were examined individually and all three single-cells showed the same sex chromosome anomaly (data in Supplementary Figure S1).

The cell-based NIPT result was reported to the couple by a clinical geneticists (author IV) and invasive testing was recommended. The woman subsequently attended the first trimester screening at GA 12 + 5 where the nuchal translucencies were 1.2 and 1.3 mm, PAPP-A 0.514 MoM and fβ-hCG 0.34 MoM, which gave combined risks for trisomy 21 of 1:1,077 and 1:917, respectively. Due to the cell-based NIPT result, confirmatory prenatal diagnosis using invasive sampling from both placentae was carried out at GA 13 + 1. The invasive prenatal analysis indicated that the twins were monozygotic and demonstrated that both fetuses had the karyotype arr(X)x2,(Y)x1 (data not shown), in accordance with the findings of *cell-based* NIPT, Figure 2. This result was reported to the couple in a genetic counselling session in a public setting.

**FIGURE 3:**
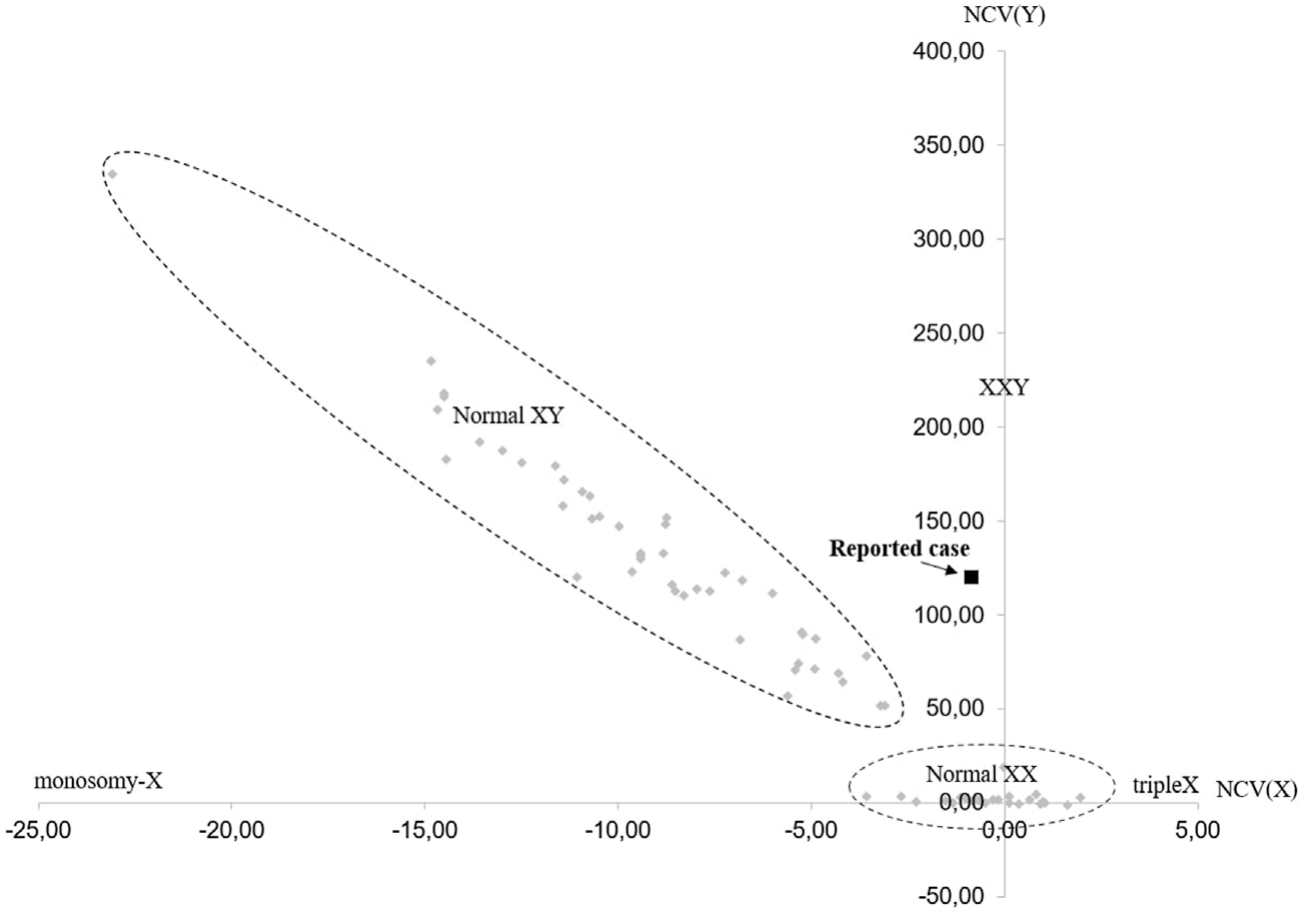
Cell-free NIPT result for chromosome X and chromosome Y. The horizontal axis represents the normalized level of X-chromosomal material [NCV(X)], and the vertical axis represents level of Y-chromosomal material detected [NCV(Y)]. The normal range for female euploid samples (Normal XX) and male euploid samples (Normal XY) are marked by the dotted lines. The cell-free NIPT result for the reported case is marked with a black square. The cell-free NIPT result suggests a 47,XXY karyotype in at least one of the fetuses. NCV = Normalized Chromosome Value (for chromosome X and chromosome Y, respectively).

For research purposes, cell-free NIPT was conducted retrospectively, after invasive testing had confirmed the cell-based NIPT result. The fetal fraction was 8% and the result showed increased risk of 47,XXY in at least one of the fetuses and with two copies of chromosome 13, 18 and 21 (Figure 3).

## Discussion

We report the detection of a sex chromosome anomaly (Klinefelter Syndrome) in a dichorionic twin pregnancy, using circulating fetal cells (extravillous trophoblasts) for NIPT. Twenty fetal cells were isolated from the maternal blood sample and STR analysis showed that they all shared the same genetic profile. This highly increased the likelihood of a pregnancy with monozygotic twins, although the presence of dizygotic twins could not be excluded. The finding of 47,XXY in circulating fetal cells indicated that at least one of the fetuses would develop Klinefelter Syndrome, but also that the pregnancy most likely was a monozygotic pregnancy with both fetuses affected. Invasive testing confirmed the sex chromosome anomaly and that both fetuses were affected.

The present case shows how noninvasive testing options, and in particular cell-based NIPT, can provide valuable information to prenatal diagnosis of multiple pregnancies. As always, the test limitations need to be communicated during genetic counseling and positive NIPT results must be confirmed invasively. The clinical applicability of cell-based NIPT is yet to be established in multiple pregnancies, however, the establishment of cell-based NIPT for detecting aneuploidies and CNVs in singleton pregnancies is already published (Breman et al., 2016; Kølvraa et al., 2016; Petersen et al., 2017; Vossaert et al., 2018; Vossaert et al., 2019; Hatt et al., 2020) and has been commercialized (www.evitatest.com).

Specifically regarding multiple pregnancies, the utility of cell-free NIPT to screen for the common trisomies has been shown (Fosler et al., 2017; Gil et al., 2019; Motevasselian et al., 2020). However, there are some challenges when performing cell-free NIPT in twin pregnancies but these varies between the genetic testing methods. Cell-free NIPT by low coverage massive parallel sequencing (MPS) is currently used in the Central Denmark Region and differs from the SNP-based approaches. For both testing methods, a reliable cell-free NIPT result depends on an adequate fetal fraction, i.e., the amount of cell-free DNA (cfDNA) that derives from the fetus(es) in the background of maternal cfDNA. A recent study using a SNP-based approach for analysis of cffDNA found that the fetal fraction in twin pregnancies is generally higher than in singleton pregnancies, but the contribution from each fetus, however, is lower (Hedriana et al., 2020). Moreover, analysis of dizygotic twin pregnancies showed large differences in the contribution of cffDNA from each of the fetuses (Hedriana et al., 2020). Therefore, the authors concluded that the utility of cell-free NIPT as a screening tool in twin pregnancies requires information on both the zygosity and the discrete fetal fractions (Hedriana et al., 2020). Importantly, this information cannot be obtained from the currently used cell-free NIPT technology based on MPS.

For cell-based NIPT, multiple pregnancies constitute some of the same challenges as has been described for cell-free NIPT but testing of circulating fetal cells has some strengths. In dizygotic twin pregnancies, it should be possible to isolate circulating fetal cells from both fetuses to obtain individual STR profiles and thus NIPT results. In monozygotic twin pregnancies, as in the present case, the identification and testing of multiple fetal cells with identical profiles will increase the probability that both fetuses have been tested. However, dizygotic twins can still not be excluded as the contribution of circulating fetal cells may solely originate from one twin. Invasive confirmatory testing is needed to obtain the prenatal diagnosis. More research is needed to validate the use of cell-based NIPT in twin pregnancies, but cell-based NIPT may contribute with valuable genetic information for couples who restrain or hesitate from invasive testing.

The positive predictive value (PPV) for cell-free NIPT for SCAs is low (overall PPV for SCAs is 38.9%) compared to the PPV for common trisomies and false positive results may lead to unnecessary invasive testing (Lüthgens et al., 2021). Klinefelter syndrome is the most common SCA in males affecting in 500 live male births (Kanakis and Nieschlag, 20182018) and the PPV for cell-free NIPT finding of 47,XXY has been estimated to range between 70 and 90% in singleton pregnancies (Wang et al., ; Samango-Sprouse et al., 2017). The PPV for Klinefelter syndrome in twin pregnancies is unknown. In a recent study, Ronzoni et al. performed prenatal or postnatal karyotyping after cell-free NIPT suggested an increased risk of 47,XXY in 34 pregnancies (Ronzoni et al., 2021). In nine cases the cell-free NIPT result was not confirmed and in one third of these cases the fetus had a normal 46,XY karyotype corresponding to a false-positive rate of 8.8%. In general, false-positive cell-free NIPT results can be caused by maternal factors such as copy number variants, mosaic SCAs or malignancy and maternal DNA sequencing is recommended to avoid redundant invasive testing (Wang et al., 2015; Hartwig et al., 2018). Oppositely, these maternal factors do not interfere with the data interpretation when the starting material is intact circulating fetal cells.

In the study by Ronzoni et al., four cases presented with mosaic forms of SCAs in the pre- or postnatal follow-up after cell-free NIPT showed an increased risk of Klinefelter syndrome in the fetus (Ronzoni et al., 2021). Various grades of mosaicism (47,XXY/46,XY) is known to be present in 10–20% of Klinefelter males and these individuals tend to present with a milder form of the syndrome (Kanakis and Nieschlag, 20182018). A prenatal discovery of high-grade mosaicism by cell-based NIPT has previously been reported (Vestergaard et al., 2017), but more data is needed to determine the detection rate of mosaicism, which is expected to increase with the mosaic grade and the number of individually analyzed circulating fetal cells. However, mosaicism may be overlooked in cases where only few circulating fetal cells are harvested. Therefore, detection of mosaicism is likely to be a weakness of cell-based NIPT, especially if low-grade placental mosaicism is a requested result. In the present case, neither of the NIPT results (based on circulating fetal cells and cffDNA) indicated mosaicism in the fetuses and a non-mosaic 47,XXY karyotype was confirmed invasively by CVS from both placentae.

In Denmark, cell-free NIPT is a free-of-charge offer as an alternative to invasive testing if the first trimester risk assessment for Downs Syndrome is above 1:300. The pregnant woman in the present case received a low-risk result and was therefore not offered prenatal testing in the public setting. As the couple at an early gestational age were interested in reducing the risk of aneuploidy and CNVs, they had already opted for cell-based NIPT at a private clinic prior to the first trimester screening. This case shows how noninvasive testing options can provide significant information on the genetic status of fetus(es) for parents-to-be even before the first trimester screening. Increased use of NIPT early in pregnancy emphasize the need for new approaches for prenatal counseling before the first trimester risk assessment and optimally the available data should be integrated in the risk assessment algorithm if the result is normal. This would significantly reduce the residual risk of fetal aberrations and likely improve both prenatal care and parental anxiety both in case of negative and positive findings. In conclusion, this case report shows how cell-based NIPT demonstrated Klinefelter Syndrome at an early gestational age and thereby provided significant information for the decision-making to a couple expecting twins.

## Data Availability

The original contributions presented in the study are included in the article/Supplementary Material, further inquiries can be directed to the corresponding author.
