# Supplementary figures and images for: Cell-Based NIPT Detects 47,XXY Genotype in a Twin Pregnancy
Source: Front Genet. 2022 Mar 11;13:842092. doi: 10.3389/fgene.2022.842092 (PMC8963804; doi:10.3389/fgene.2022.842092)

## Slide 1
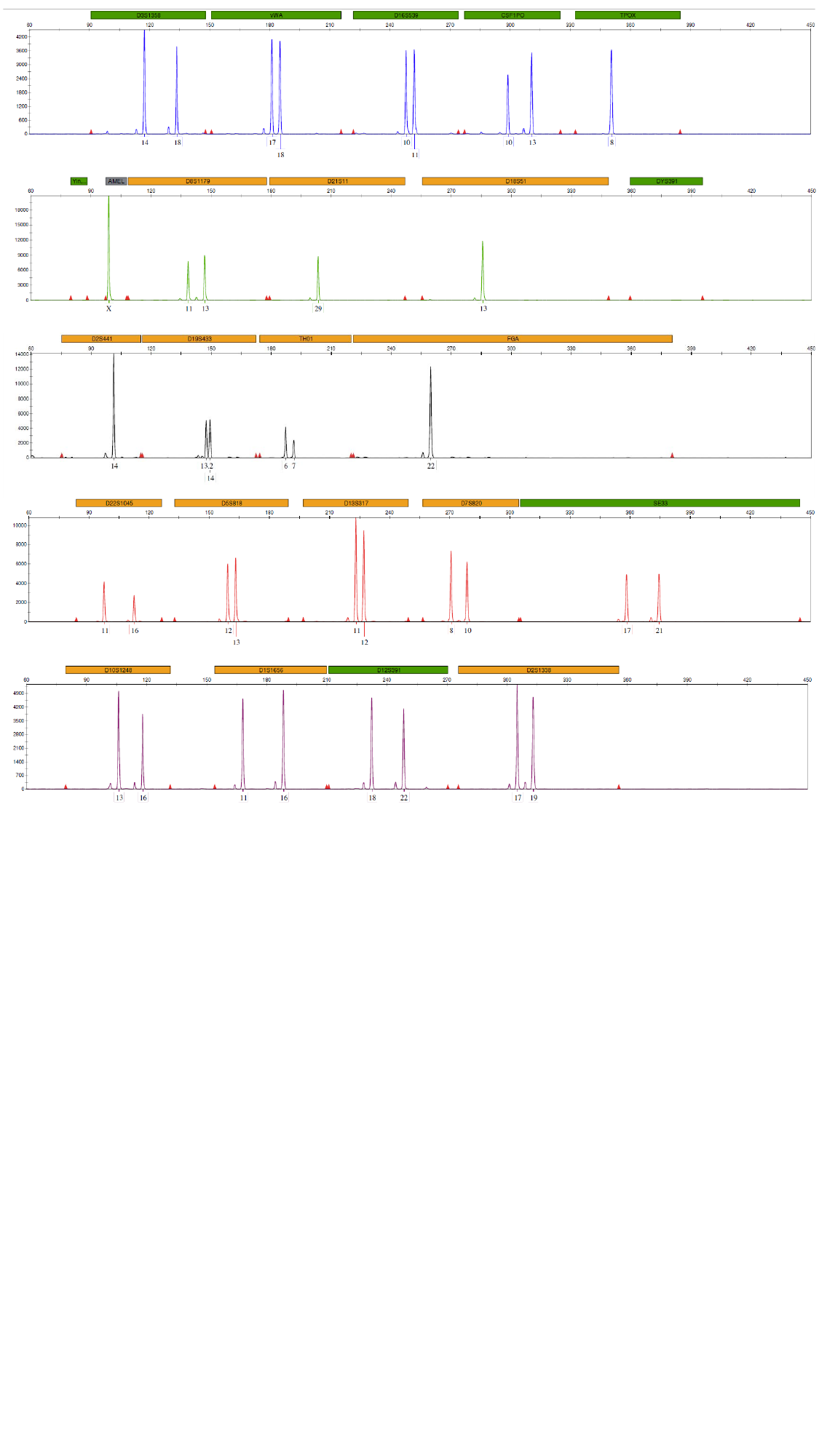

## Slide 2
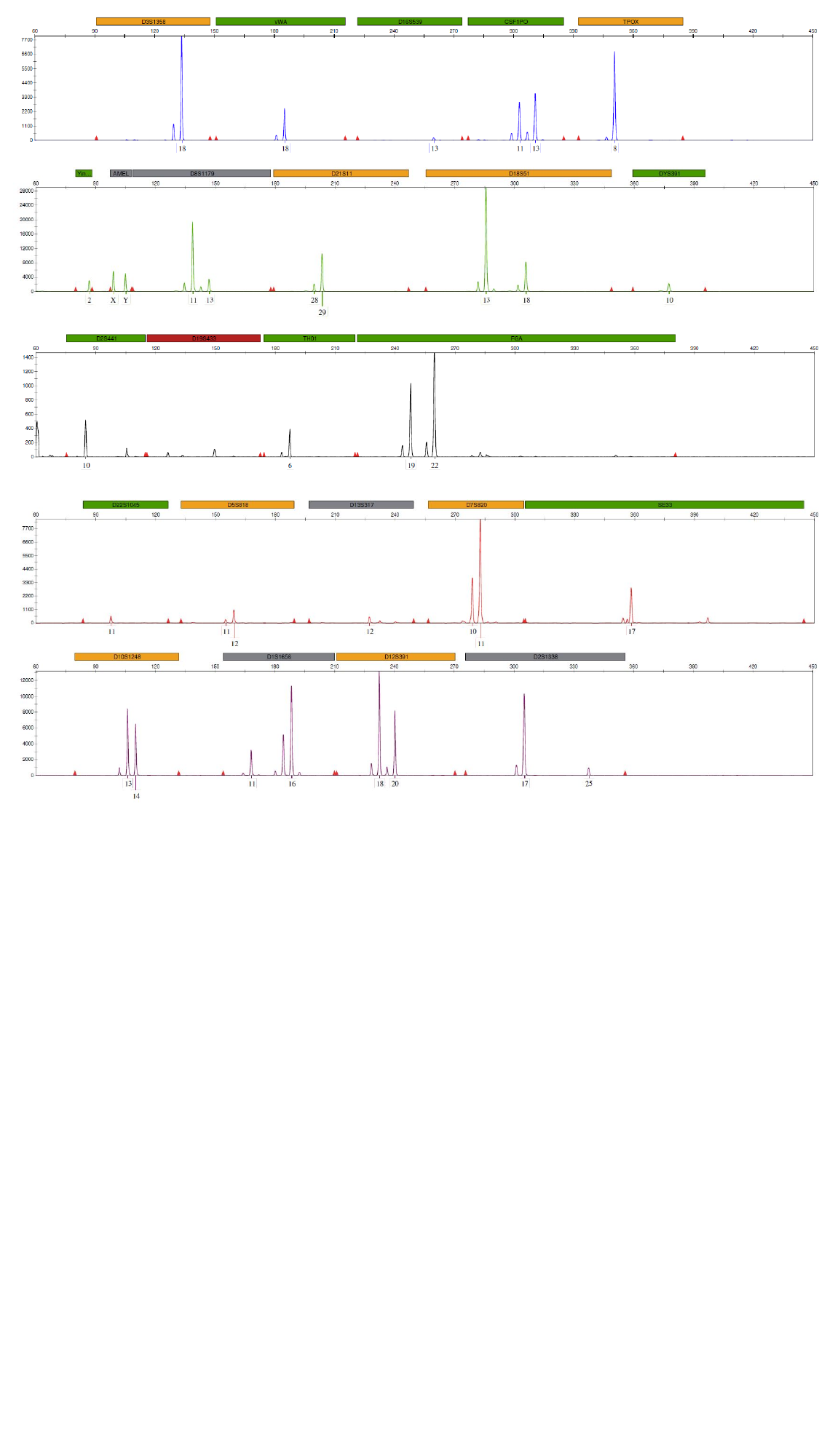

Supplement: Supplementary file 1 [file Presentation1.pptx]
